# Proposal and validation of an angular External Fixation Index (aEFI) for standardized reporting of gradual angular deformity correction: a time-normalized descriptive metric

**DOI:** 10.1007/s00264-026-06779-2

**Published:** 2026-04-02

**Authors:** Mohamed Sayed ELazab, Mohammed Ahmed Moussa, Ahmed Mahmoud Fouad Elguindy

**Affiliations:** Fayoum University, Al Fayyum, Egypt

**Keywords:** Genu varum, Circular external fixation, Angular deformity correction, External fixation index, Distraction osteogenesis

## Abstract

**Background:**

​ Gradual correction of genu varum using circular external fixators is well-established. Although the External Fixation Index (EFI) is widely used in linear bone lengthening, no standardized, time-normalized metric exists for angular deformity correction. This study introduces the angular External Fixation Index (aEFI) as a descriptive tool and evaluates internal consistency within a clinical cohort treated with oblique proximal tibial corticotomy (OPTC).

**Methods:**

​ A prospective cohort study included 22 patients (30 knees) who underwent gradual genu varum correction using OPTC and circular external fixation. The aEFI was calculated as total duration of external fixation (weeks) divided by achieved angular correction (degrees). Radiographic evaluation included medial proximal tibial angle (MPTA) and hip–knee–ankle angle measured preoperatively and at final follow-up. Functional outcomes were assessed using the Western Ontario and McMaster Universities Osteoarthritis Index, and the Stanmore Limb Reconstruction Score.

**Results:**

At a mean follow-up of 16.5 ± 3.25 months, coronal alignment was restored in all knees. Mean fixation duration was 27.4 ± 6.2 weeks, with a mean aEFI of 1.89 ± 0.41 weeks/degree. An inverse association was observed between correction magnitude and aEFI (r = -0.88, *p* < 0.001), reflecting the reduced proportional effect of fixed treatment phases with larger corrections. Functional scores improved, and minor pin-tract infections occurred in 20% of knees and resolved conservatively.

**Conclusion:**

​ The proposed aEFI serves as a standardized, descriptive, time-normalized metric for reporting treatment duration relative to angular correction. External validation across different constructs and deformity patterns is warranted.

## Introduction

​Genu varum correction with circular external fixation represents a well-established approach for managing high-magnitude deformities. Despite the widespread acceptance of this technique, reporting of treatment duration remains non-standardized, limiting meaningful comparisons across different surgical approaches and fixation constructs. In 1987, De Bastiani et al. introduced the External Fixation Index (EFI) for linear lengthening [1], a landmark metric that enabled objective comparisons across lengthening techniques by normalizing treatment duration to the amount of length achieved. For angular corrections, however, no equivalent time-normalized metric exists, despite the frequent use of articulated hinges and sophisticated external fixation systems [2]. This methodological gap hinders the ability to compare outcomes across centres and to evaluate the relative efficiency of different correction techniques [3].

To address this deficiency, we propose an angular External Fixation Index (aEFI) defined as weeks in frame per degree of angular correction. This descriptive index is intended to standardize reporting of treatment burden in angular distraction osteogenesis, complementing rather than replacing established deformity planning principles [4, 5]. The aEFI is not designed as a surrogate marker of biological healing but rather as a tool for quantifying the temporal investment required to achieve a given correction.

To illustrate the application and validation of this proposed metric, we utilize a cohort of patients treated with oblique proximal tibial corticotomy (OPTC) and circular external fixation for gradual genu varum correction.

The primary objectives of this study are: (1) to propose the aEFI as a descriptive, time-normalized metric for angular correction, (2) to assess its internal consistency within a clinical cohort, and (3) to discuss its potential applications and limitations in orthopaedic deformity research.

### ​​patients and methods

A prospective study was conducted on 22 patients (30 knees) who underwent joint-preserving deformity correction for genu varum by a single senior surgeon. All patients were ≥ 15 years old and exhibited symptomatic coronal plane malalignment. Exclusion criteria included advanced knee osteoarthritis, metabolic bone disease, and prior proximal tibial osteotomy. The study was approved by the institutional review board (R 475,107), and written informed consent was obtained from all patients.

Preoperative evaluation included clinical examination and standardized full-length standing radiographs (anteroposterior and lateral views). Radiographic assessment included hip–knee–ankle (HKA) angle, medial proximal tibial angle (MPTA), mechanical lateral distal femoral angle (mLDFA), joint line convergence angle (JCA), and posterior tibial slope (PTS) angle. Radiographic measurements were performed digitally using Philips EBW 2.1 software (Philips Healthcare, San Jose, California, USA). Measurements were obtained independently on preoperative and postoperative radiographs by two blinded observers to minimize measurement bias.

### Surgical technique

**Fig. 1:**
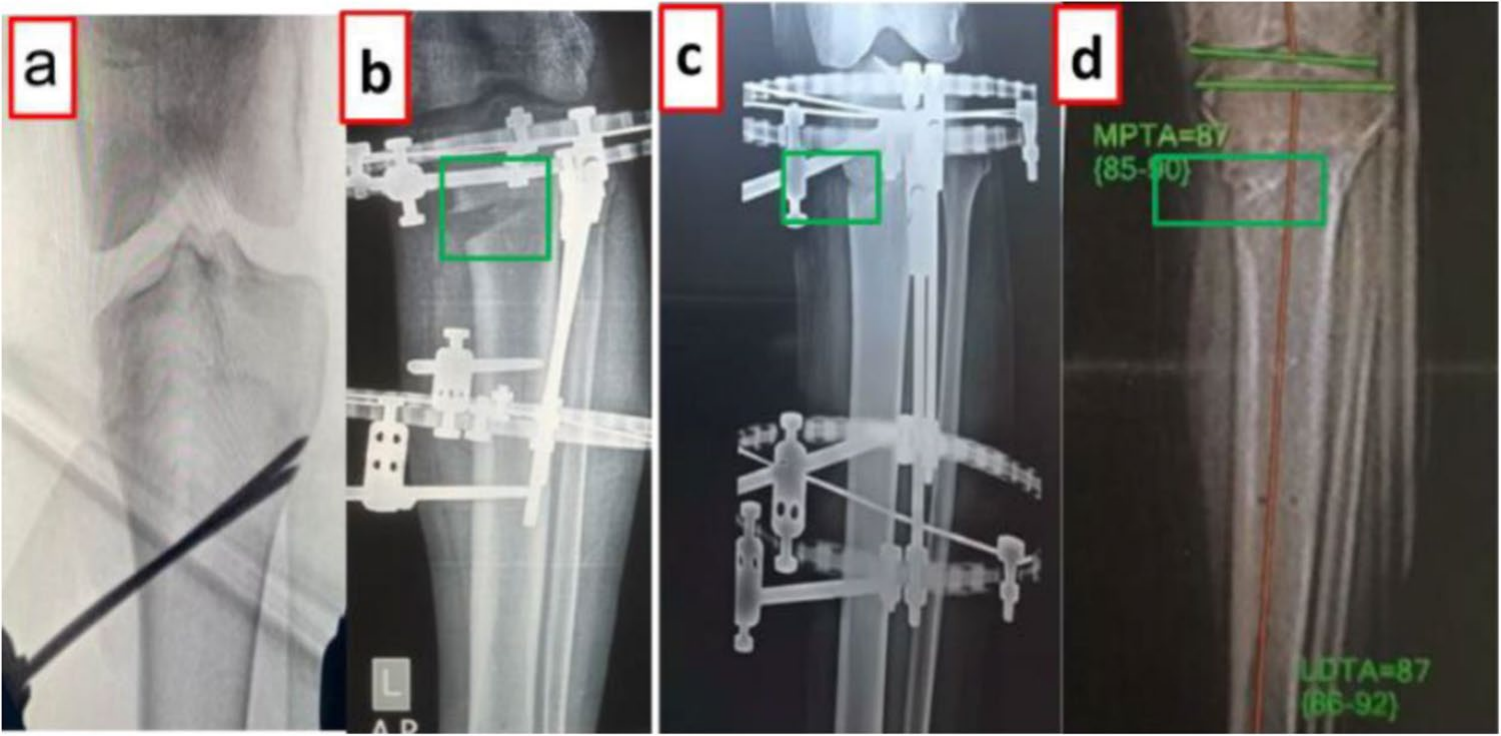
Schematic representation of the oblique proximal tibial corticotomy and gradual distraction process. **a** initial surgical stage: placement of 2 parallel k-wires obliquely from medial cortex of proximal tibia toward fibular head to guide oblique corticotomy, followed by multiple drilling, then an osteotome was used to connect drilling holes. **b** Distraction phase**:** gradua**l** distraction at corticotomy site (green box) by Ilizarov external fixator. **c** Consolidation phase: development of the bone regenerate (green box) at corticotomy site. **d** Final result: restored mechanical axis (MPTA) and complete osseous consolidation (green box) following frame removal

Under general or regional anaesthesia and fluoroscopic guidance, an oblique proximal tibial corticotomy was performed. Through a limited medial incision distal to tibial tuberosity, two parallel k-wires were inserted from medial cortex obliquely toward head of fibula to guide the corticotomy **(**Fig. 1a**)**. Multiple drill holes were created along this trajectory using a 4.5 mm drill bit and the corticotomy was completed using an osteotome. Then, osteoclasis was achieved by controlled valgus manipulation. Preservation of the periosteum and lateral cortex was emphasized to optimize regenerate biology and mechanical stability.

A circular external fixator was applied, consisting of one proximal and one distal ring aligned to the tibial mechanical axis. Each ring was secured using three to four fixation points (wires, half pins). Opposing anterolateral and posterolateral hinges were aligned in the coronal plane at the level of the lateral cortex of the corticotomy, and placement of motor rod medially to allow gradual valgus correction. Distraction was initiated after a latency period of seven days at approximately 0.25 turn every six h and adjusted according to clinical and radiographic findings **(**Fig. 1b**)**. Partial to full weight bearing was encouraged throughout treatment. The consolidation phase was defined by the appearance of bridging callus across at least three cortices on anteroposterior and lateral radiographs **(**Fig. 1c**)**. The frame was removed once solid osseous consolidation was confirmed clinically and radiographically (Fig. 1d**)**.

### Outcome measures

The primary outcome measures were radiographic correction (MPTA, HKA).

Time in frame was calculated as the total duration of external fixation from application until removal.

The aEFI was defined as total time in external fixation (weeks) divided by the total angular correction (degrees)

$$\mathrm{aEFI} \, (\mathrm{weeks} /\mathrm{degree}) \; = \;\frac{Total\ Duration\ in\ Fixator\ (weeks)}{total\ degrees\ of\ correction\ (\Delta MPTA)}$$

The MPTA was selected to reflect site-specific correction at the corticotomy level and minimize confounding from femoral or compensatory deformities.

Secondary outcomes included functional scores and complications. Functional outcome was assessed using the Western Ontario and McMaster Universities Osteoarthritis Index (WOMAC) [6] preoperatively and at final follow-up after frame removal. Additionally, the Stanmore Limb Reconstruction Score (SLRS) [7] was recorded at final follow-up to evaluate treatment burden, psychological wellbeing, and social integration – domains particularly relevant to patients undergoing prolonged external fixation.

## Statistical analysis

​Statistical analysis was performed using SPSS version 26.0 (IBM Corp., Armonk, NY, USA). Normality of data distribution for all continuous variables was evaluated using the Shapiro–Wilk test. For data following a normal distribution, the paired-sample t-test was employed to compare preoperative and postoperative values for all radiographic and functional outcome measures. In instances where the data did not meet the criteria for normality, the Wilcoxon signed-rank test was utilized as a non-parametric alternative. To evaluate differences across the three deformity magnitude subgroups (mild, moderate, and severe), a one-way ANOVA was conducted. Deformity magnitude subgroups were defined based on the total angular correction (∆MPTA): mild (< 10°), moderate (10°−20°), severe (> 20°). The strength and direction of the linear relationship between variables were quantified using Pearson’s correlation coefficient (r). Scatter plot visualization was used to illustrate the relationship, allowing visual assessment of data distribution and linear trend. The intraclass correlation coefficient (ICC) was calculated using a two-way random-effects model with absolute agreement. An ICC value greater than 0.75 was considered indicative of good reliability. A multiple linear regression model was conducted to identify independent predictors (age, BMI, ∆MPTA). Quantitative data were summarized as mean ± standard deviation (SD) and range, with a p-value < 0.05 considered statistically significant.

## Results

The cohort consisted of 22 patients (30 knees). The mean age was 22.3 ± 7.02 years (range: 15–38 years). The mean BMI (kg/m^2^) was 31.1 ± 5.8 (range: 20–39).

The mean duration of external fixation was 27.4 ± 6.2 weeks (range: 18–42 weeks).

Radiographic and clinical measurements demonstrated excellent intra-observer reliability (ICC = 0.93; 95% CI: 0.88–0.96) and inter-observer reliability (ICC = 0.90; 95% CI: 0.85–0.94). These values indicate a high level of consistency and reproducibility.

Restoration of coronal alignment was achieved in all treated knees (Table 1), with the mechanical axis corrected to within physiological limits (*p* < 0.001).

**Table 1:** Radiographic alignment parameters (*N* = 30)

Parameter	Preoperative (Mean ± SD, Range)	Postoperative (Mean ± SD, Range)	∆ (Correction)	*P*-value
HKA Angle (^⸰^)	154.39 ± 9.46 (140–169)	175.72 ± 3.63 (170–183)	21.33 ± 9.15	< 0.001
MPTA (^⸰^)	73.55 ± 5.91 (63–80)	88.45 ± 2.45 (85–95)	14.90 ± 5.50	< 0.001

*HKA* hip-knee-ankle, *MPTA* medial proximal tibial angle, *SD* standard deviation, *p*-value < 0.001 is highly statistically significant

The mean MPTA improved significantly from 73.55° ± 5.91° preoperatively to 88.45° ± 2.45° postoperatively, within a mean correction of 14.9º ± 5.5º (range: 7º—26º, *p* < 0.001). Sagittal plane parameter (PTS) was preserved, with no significant change (13.5º ± 7.0º pre-op vs. 13.7º ± 6.8º post-op).

**Fig. 2:**
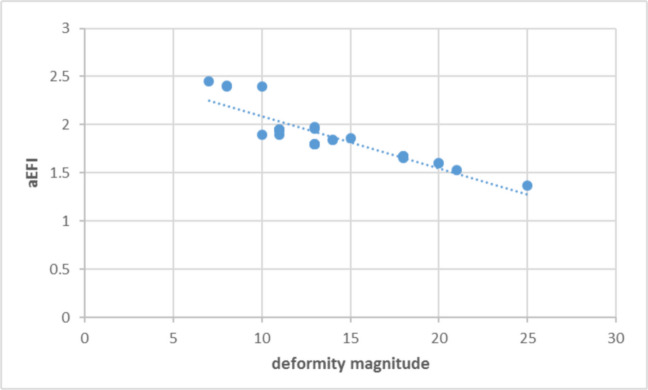
Scatter plot illustrating the relationship between angular deformity magnitude (degrees of correction) and the angular External Fixation Index (aEFI). An inverse linear trend is observed, with lower aEFI values associated with larger angular corrections. This visualization illustrates the observed correlation within the cohort

The mean aEFI for entire cohort was 1.89 ± 0.41 weeks/degree of correction (Table 2). An inverse linear association was observed between correction magnitude and aEFI (r = −0.88, *p* < 0.001) with lower aEFI values noted in knees undergoing larger angular corrections. This trend is visually represented in the scatter plot (Fig. 2).

**Table 2:** The relationship between deformity magnitude and aEFI

Correction magnitude	N (knees)	Time in frame (weeks)	aEFI (weeks/degree)
Mild (< 10º)	7	19.8 ± 2.5	2.48 ± 0.31
Moderate (10º- 20º)	14	26.5 ± 3.8	1.83 ± 0.22
Severe (> 20º)	9	34.5 ± 4.2	1.51 ± 0.18
Overall Mean	30	27.4 ± 6.2	1.89 ± 0.41

*aEFI* angular External Fixation Index, *SD* standard deviation, *p* < 0.001

An exploratory multiple linear regression model showed that ∆MPTA was the only significant predictor of aEFI (β = −0.84, *p* < *0.001)*. Patient age (*p* = *0.48)* and BMI (*p* = *0.64)* were not significantly associated with aEFI within this cohort.

At a mean follow-up of 16.5 ± 3.25 months (range: 10–23 months), radiographs confirmed complete osseous consolidation at the corticotomy site in all cases, with no loss of correction observed.

Functional outcomes improved substantially. The mean WOMAC score decreased from 58 ± 8 preoperatively to 18 ± 6 at final follow-up (*p* < 0.001), reflecting improvement in pain and stiffness alongside improved physical function.

The mean SLRS at final follow-up was 82.4 ± 7.6, reflecting a favourable overall treatment experience.

Pin-tract infection was the most frequently observed complication, occurring in six of 30 knees (20%). All cases were managed successfully with local pin care and oral antibiotics. No cases of deep infection, delayed-union, non-union, premature consolidation, mechanical fixator failure or loss of correction were recorded.

## Discussion

This study introduces the aEFI as a descriptive, time-normalized metric for reporting treatment duration in gradual angular correction. Using a cohort of patients treated with OPTC and circular external fixation, we demonstrate the calculation of this index and examine cohort-based internal evaluation. In this cohort, the mean aEFI was 1.89 weeks/degree and may provide a reference value for this specific technique and construct, but the primary contribution of this work is methodological: The aEFI offers a standardized language for reporting treatment burden per unit of angular correction [3].

The absence of a standardized temporal metric for angular correction represents a significant gap in the deformity correction literature. Without such a tool, meaningful comparisons across studies remain challenging. For example, one centre might report a mean frame time of 12 weeks for a 15° correction while another reports 16 weeks for an identical magnitude; without a normalized index, the contributions of surgical technique, frame configuration, and patient factors cannot be effectively disentangled [3]. The aEFI addresses this gap by enabling researchers to report, compare, and ultimately meta-analyze treatment burden across studies. In this respect, it serves a purpose analogous to the EFI in limb lengthening, which fundamentally transformed comparative research in that domain [1].

The selection of MPTA as the primary radiographic parameter for aEFI calculation merits justification. MPTA provides site-specific accuracy in reflecting deformity at the proximal tibia and correction achieved at the corticotomy level [8]. Relying solely on global mechanical axis measurements can be misleading, as these may be influenced by compensatory mechanisms or unrecognized femoral deformities, potentially leading to iatrogenic joint line obliquity [4, 9]. Recent work has highlighted the risks of tibial overcorrection when these principles are not followed [10]. The excellent inter-observer reliability (ICC = 0.90) and intra-observer reliability (ICC = 0.93) observed for the MPTA measurements support the reproducibility of the aEFI when derived from standardized radiographic assessment.

A notable finding of this study was the strong inverse correlation between correction magnitude and aEFI (r = −0.88, *p* < 0.001). While statistically robust, this observation must be interpreted correctly: it is not a biological discovery but rather a mathematical consequence of fixed treatment phases. The latency period (7 days) and consolidation phase (approximately 4–6 weeks) are largely independent of the amount of angular correction achieved. When these fixed time components are divided by a larger number of angular degrees, the resulting index necessarily decreases. This dilution effect has been previously documented in the linear distraction literature [11, 12] and is now explicitly acknowledged for angular correction. Clinicians and researchers should therefore interpret lower aEFI values in larger corrections as a reflection of geometric scaling rather than as evidence of accelerated biological healing.

From a clinical perspective, the aEFI may serve as an adjunctive reference for patient counseling and expectation management, when interpreted within cohort-specific limitations. Reference values derived from a defined technique and fixation construct may assist surgeons in providing individualized, experience-based estimates of treatment duration, particularly in limb reconstruction settings where prolonged external fixation and treatment burden can influence patient compliance and psychological well-being [13]. The aEFI is not intended to replace established alignment parameters but rather to complement existing planning tools.

The significant improvements observed in WOMAC scores confirm that the radiographic corrections achieved were clinically meaningful, with patients experiencing substantial reductions in pain and stiffness alongside enhanced physical function. The SLRS of 82.4 ± 7.6 indicates that patients found the treatment burden acceptable despite prolonged external fixation, a finding consistent with previously reported validation data [7]. The SLRS is particularly relevant as it directly addresses the subjective experience of the time in frame that the aEFI quantifies objectively.

The observed complication rate (20% pin-tract infections, all resolving with conservative management) aligns with published literature on circular external fixation [14, 15]. The absence of major complications such as deep infection, non-union, or mechanical failure supports the short-term safety profile of the described approach within this cohort.

This study has several limitations that should be acknowledged. Although the prospective design strengthens internal validity, the single-center, single-surgeon design results in a relatively small cohort, which may constrain the broader applicability of these results. In addition, the regression model may overestimate effect size, and the findings should be interpreted as exploratory rather than predictive. The absence of a direct comparative control group limits any inference regarding the surgical technique. The aEFI is dependent on accurate angular measurements, particularly MPTA, and therefore may be influenced by radiographic positioning and measurement variability; however, the excellent inter-observer reliability (ICC = 0.90) supports the reproducibility of these measurements. Finally, while a strong correlation was observed between deformity magnitude and aEFI, this relationship reflects internal consistency within a single cohort rather than external validation. The follow-up period, while adequate to assess consolidation, is relatively short for detecting late complications or recurrence. Longer-term surveillance is warranted. Larger multicenter studies with comparative designs are required to further validate the aEFI and to establish its applicability across different deformity patterns and fixation constructs (e.g., Taylor Spatial Frame, hexapod systems).

## Conclusion

The aEFI is proposed as a descriptive, time-normalized metric for reporting treatment duration in gradual angular correction. Internal consistency was demonstrated within this cohort, and the observed inverse correlation with correction magnitude is a mathematical consequence of fixed treatment phases. While further external validation is required, the aEFI may serve as a useful adjunct for internal comparison, surgical planning, and patient counseling within a specific surgical protocol.

## Data Availability

The data are available from the corresponding author upon reasonable request.
